# Macroscopic and microscopic spatially-resolved analysis of food contaminants and constituents using laser-ablation electrospray ionization mass spectrometry imaging

**DOI:** 10.1007/s00216-014-7948-8

**Published:** 2014-06-25

**Authors:** Michel W. F. Nielen, Teris A. van Beek

**Affiliations:** 1 RIKILT Wageningen UR, P.O. Box 230, 6700 AE Wageningen, The Netherlands; 2 Wageningen University, Laboratory of Organic Chemistry, Dreijenplein 8, 6703 HB Wageningen, The Netherlands

**Keywords:** Mass spectrometry, Imaging, LAESI, Laser ablation, Food analysis, Agriforensics, Mycotoxins, Pesticides, Glycoalkaloids

## Abstract

Laser-ablation electrospray ionization (LAESI) mass spectrometry imaging (MSI) does not require very flat surfaces, high-precision sample preparation, or the addition of matrix. Because of these features, LAESI-MSI may be the method of choice for spatially-resolved food analysis. In this work, LAESI time-of-flight MSI was investigated for macroscopic and microscopic imaging of pesticides, mycotoxins, and plant metabolites on rose leaves, orange and lemon fruit, ergot bodies, cherry tomatoes, and maize kernels. Accurate mass ion-map data were acquired at sampling locations with an *x*–*y* center-to-center distance of 0.2–1.0 mm and were superimposed onto co-registered optical images. The spatially-resolved ion maps of pesticides on rose leaves suggest co-application of registered and banned pesticides. Ion maps of the fungicide imazalil reveal that this compound is only localized on the peel of citrus fruit. However, according to three-dimensional LAESI-MSI the penetration depth of imazalil into the peel has significant local variation. Ion maps of different plant alkaloids on ergot bodies from rye reveal co-localization in accordance with expectations. The feasibility of using untargeted MSI for food analysis was revealed by ion maps of plant metabolites in cherry tomatoes and maize-kernel slices. For tomatoes, traveling-wave ion mobility (TWIM) was used to discriminate between different lycoperoside glycoalkaloid isomers; for maize quadrupole time-of-flight tandem mass spectrometry (MS–MS) was successfully used to elucidate the structure of a localized unknown. It is envisaged that LAESI-MSI will contribute to future research in food science, agriforensics, and plant metabolomics.

**Electronic supplementary material:**

The online version of this article (doi:10.1007/s00216-014-7948-8) contains supplementary material, which is available to authorized users.

**Figure:**
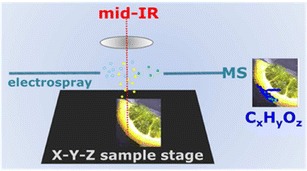
ᅟ

## Introduction

In quality and safety assessment the chemical composition of foods and food ingredients is usually determined in an averaged dimension only. Typically, the sample material is extracted before further instrumental analysis by, e.g., gas or liquid chromatography coupled to mass spectrometry (GC–MS or LC–MS), thereby sacrificing any spatial chemical information. Spatially-resolved food analysis is not only of interest to scientists searching for added-value natural food ingredients but also has applications in food quality and safety; for example, to investigate the presence and/or degree of surface contamination or surface defects or to search for early-ripening markers. It is also important in agriforensics, to investigate the use of banned substances in the food chain, and in food security, where information is needed about the options for use and/or reuse of off-spec food and feed ingredients (highly relevant in the future to feed a growing world population). However, two-dimensional (2D) and three-dimensional (3D) spatially-resolved analysis in the conventional manner requires sectioning, extraction, sample preparation, instrumental analysis of all the individual sections produced, and reconstruction of the data thus obtained for visualization of 2D and 3D images. This is an extremely laborious and time-consuming process and, as a consequence, not often used.

Mass spectrometry imaging (MSI) is developing rapidly as a tool for 2D and 3D spatially-resolved chemical-composition analysis [1–4]. Standard ionization techniques in MSI are secondary-ion mass spectrometry (SIMS) and matrix-assisted laser desorption/ionization (MALDI), both performed under vacuum conditions. The spatial resolution that can be routinely obtained is in the range of sub-μm (SIMS) to ~15 μm (MALDI). Some inherent limitations are fragmentation in SIMS and matrix interference in MALDI, which hinder the analysis of large and of small molecules, respectively [2]. More recently MSI under ambient conditions was introduced, featuring ionization techniques including desorption electrospray ionization (DESI), nano-DESI, electrospray laser-desorption ionization (ELDI), laser-induced acoustic-desorption electrospray ionization (LIAD-ESI), matrix-assisted laser-desorption electrospray ionization (MALDESI), probe electrospray ionization (PESI), sheath-flow-PESI (SF-PESI), laser-ablation electrospray ionization (LAESI), infrared-laser-ablation metastable-induced chemical ionization (IR-LAMICI), and many others [5–11]. So far, both vacuum and ambient MSI have been mainly used for analysis of precision-cut tissue slices, cell colonies, and plant leaf materials [1–9]. Müller et al. [12] used DESI-MSI to study chlorophyll degradation products in plant tissues, both directly and after imprinting on PTFE. Cabral et al. [13] developed a DESI-MSI method for potato sprouts, ginkgo leaves, and strawberries blotted on thin-layer chromatography (TLC) plates. Mandal et al. [10] studied pesticide residues on plant materials after different pesticide applications via SF-PESI. Bennett et al. [14] improved the signal-to-noise and image contrast in DESI-MSI of biological tissues by incorporating a differential mobility spectrometry section acting as a nested orthogonal ion filter. The limited spatial resolution of DESI-MSI (approx. 250 μm) was successfully improved to 12 μm after the introduction of nano-DESI [15]. In nano-DESI, first a surface extraction is performed in a liquid bridge formed by a solvent confined between two capillaries [15]. This is followed by standard nanoelectrospray ionization. Nano-DESI was successfully used in the sampling of microbial colonies directly from a Petri dish by performing line scans separated by 1 mm; however, the required careful *z*-axis control complicated its application [16]. A common disadvantage of SIMS, MALDI, and nanoDESI-MSI is the requirement for flat sample surfaces, which is obviously not very practical for food analysis; ELDI, IR-LAMICI, and LAESI are less demanding in this respect.

In IR-LAMICI and in LAESI the surface is probed by a mid-IR laser beam at 2,940 nm that excites the hydroxyl vibrations of water molecules in the sample [11, 17]. The neutral molecules in the ablated plume are ionized after interaction with metastable helium (or nitrogen) or with charged solvent droplets, from a nanoelectrospray ionization source, respectively. Thus desorption and ionization are decoupled, and therefore IR-LAMICI and LAESI are less demanding with respect to the sample material under investigation. The spatial resolution of the chemical images obtained is determined by the size of the ablated craters. That size depends on the laser fluence and the tensile strength of the sample, and is in the order of 200–400 μm wide and 40–50 μm deep [7, 11]. When etched fiber tips are used to deliver the laser energy, the spatial resolution can be dramatically improved, even approaching single-cell analysis [18–20]. As in DESI, reagents may be added to the solvent in LAESI (“reactive LAESI”), as revealed by Shrestha et al. [21] in the lithium cationization of lipids. The applicability of LAESI toward nonpolar analytes could be extended by heat-assisted LAESI, and the modified version was successfully used for analysis of triglycerides in avocado mesocarp [22]. Shrestha et al. [23] found more than 200 positive-ion species, including proteins, in LAESI-MSI of intact fish tissues. But despite the favorable characteristics of LAESI, (and IR-LAMICI) MSI for spatially-resolved food analysis, including a large (102 × 76 mm) temperature-controlled *x*–*y*–*z* sample stage, enabling both macroscopic (≥1 mm) and microscopic (<1 mm) analysis under ambient conditions, and the absence of any sample preparation, its applicability has been hardly investigated. In a recent LAESI-MSI study [24], the distribution of the herbicide 2,4-dichlorophenoxyacetic acid (2,4-D) was investigated on cabbage leaves, with the objective of developing the best pesticide-formulation design. According to recent ambient MS studies using direct analysis in real time (DART) ionization, a range of pesticides could be rapidly screened directly from the surface of fruit and vegetables [25, 26], but without spatial-distribution information.

In this work, fresh rose leaves, citrus fruit, apples, ergot bodies, tomatoes, and maize kernels from real-life sources are investigated by LAESI-MSI in time-of-flight (TOF)-MS mode and, for specific cases, in quadrupole TOF-MS–MS or traveling-wave ion mobility (TWIM) TOF-MS mode. The accurate mass 2D and 3D ion maps obtained clearly reveal the potential and added value of spatially-resolved targeted (pesticides, natural toxins) and untargeted food analysis.

## Materials and methods

### Reagents and sample materials

Ultra LC–MS-grade methanol and water were obtained from Actu-All Chemicals (Oss, The Netherlands). Formic acid was from Biosolve Chemicals (Valkenswaard, The Netherlands). Fresh (non-frozen) sample materials were analyzed: oranges, lemons, apples, and cherry tomatoes were purchased at a supermarket; cut roses were from a retail shop. Maize kernels and ergot bodies from rye were kindly provided by the RIKILT institute (Wageningen, The Netherlands).

### Instruments

A Protea Biosciences (Morgantown, WV, USA) model DP-1000 LAESI system was coupled to a Waters (Manchester, UK) model Synapt G2S mass spectrometer equipped with tri-wave ion-guide optics to optionally separate ions according to their ionic mobility in the gas phase. The LAESI system (Electronic Supplementary Material Figure S1) was equipped with a 2,940 nm mid-IR laser yielding a spot size of 200 μm; unless indicated otherwise, the laser was firing ten times per *x*–*y* location at 10 Hz and 100 % output energy. The system was also equipped with a syringe pump delivering a mixture of methanol–water–formic acid (50:50:0.1) at 1 μL min^−1^, a New Objective (Woburn, MA, USA) model PicoTip 5 cm × 100 μm ID stainless-steel nanospray emitter operated in the positive-ion mode at 3,800 V, and a Peltier-cooled motorized *x*–*y*–*z* sample stage (at 25 or 4 °C) scanned in serpentine mode. The sampling location *x*–*y* center-to-center distance was adjusted depending on the specific application needs. The focusing lens *L*-value and the sample stage *Z*-value were tuned for each sample with the help of the in-line camera and were typically in the order of 1.9–5.4 and 20.1–21.5 mm, respectively. The LAESI was operated using LAESI Desktop Software v.2.0.1.3 (Protea Biosciences). The TOF mass analyzer of the Synapt G2S was operated in the V-reflectron mode at a mass resolution of 18,000–20,000 (FWHM). The source temperature was 150 °C and the sampling cone voltage was 30 V (20 V in Fig. 3). The mass range acquired was *m*/*z* 100–1200 or *m*/*z* 100–1500. In QTOF-MS–MS mode the precursor-ion resolution was unity, the collision gas argon, and the trap collision energy 20 eV. In traveling-wave ion mobility (TWIM) TOF-MS mode, the ion-mobility-cell nitrogen gas flow was 90 mL min^−1^, the wave height 40 V, and the wave velocity 1,000 to 500 m s^−1^. Synapt MS and TWIM data were processed using MassLynx v4.1 SCN 883 and DriftScope v2.4 software packages, both from Waters. TOF-MS data were lock-mass corrected during data acquisition using the [(C_2_H_6_SiO)_6_ + H]^+^ impurity ion at *m*/*z* 445.1206, which was present in the nanospray background. Ions of potential interest for the generation of accurate mass ion maps were discovered via background subtraction of adjacent base-peak ion (BPI) chronogram regions from those BPI regions that coincided with the analog signal from the mid-IR laser pulses. Ion maps were created in Protea Plot v.2.0.1.3 (Protea Biosciences) after importing of MassLynx raw data files.

### Procedures

Rose leaves were directly mounted onto the *x*–*y*–*z* sample stage using Scotch Magic tape (St. Paul, MN, USA). Oranges, lemons, apples, and cherry tomatoes were sliced (thickness approximately 2–4 mm) using a kitchen knife and mounted onto the sample stage using the same tape. Ergot bodies were pressed onto a piece of adhesive gum on the sample stage. Maize kernels were sliced (thickness approximately 1 mm) using a Swiss army knife and pressed onto a piece of adhesive gum on the sample stage.

## Results and discussion

### Targeted spatially-resolved analysis of pesticide residues

**Fig. 1:**
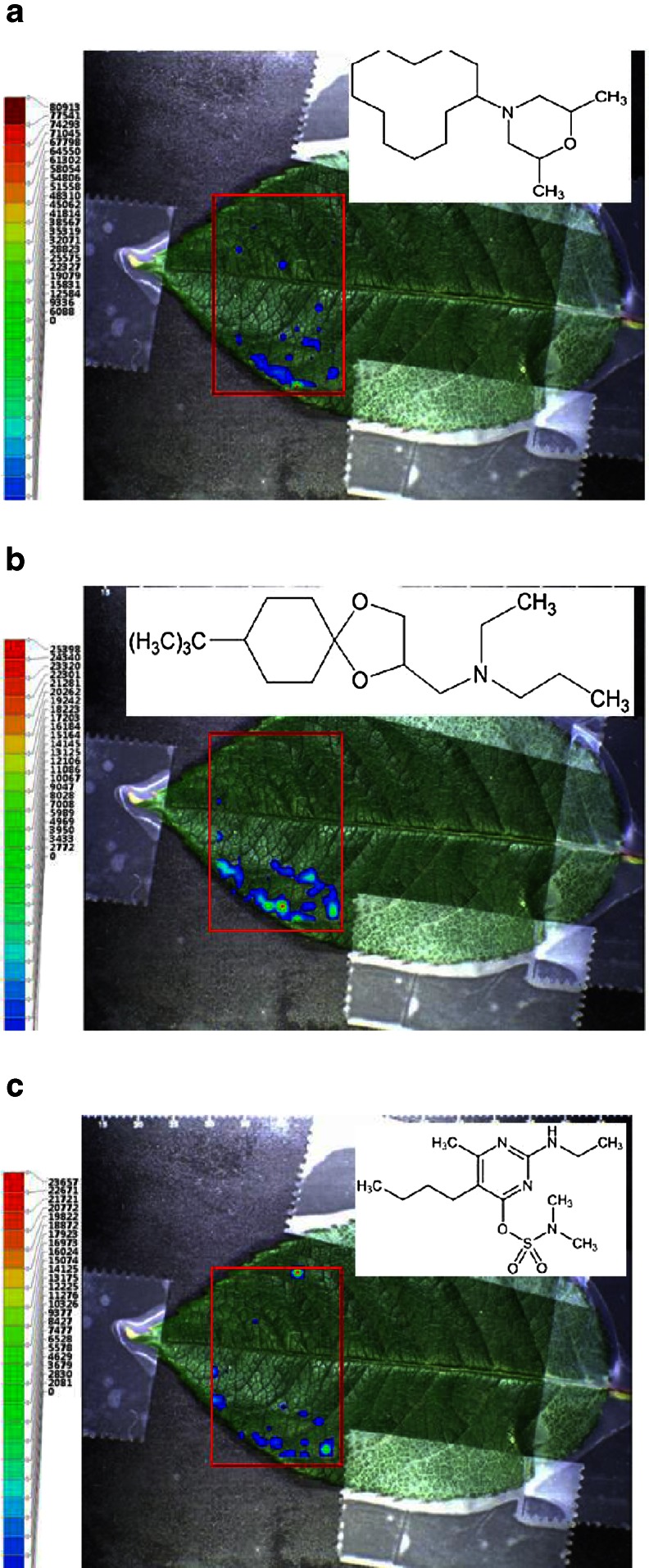
Positive-ion LAESI-TOF-MSI accurate ion maps of (**a**) *m*/*z* 282.271 (±10 mDa), (**b**) *m*/*z* 298.265 (±10 mDa), and (**c**) *m*/*z* 317.158 (±10 mDa) for a rose leaf, showing the spatial distribution of the [M+H]^+^ ions of the pesticides dodemorph, spiroxamine, and bupirimate, respectively. The *x*–*y* center-to-center sampling distance was 1 mm. The probed region is indicated by a *red rectangle*; the lowest ion intensities are not color-coded to enable visualization of the underlying features in the superimposed sample image. Other conditions, see Experimental section

From GC–MS and LC–MS analyses, cut flowers are known to contain different pesticide residues [27]. Rose leaves are usually regarded as non-food, but a few tea and yogurt recipes exist in some countries. Verification of the use of any nonregistered pesticides by rose growers is usually performed by LC–MS and GC–MS after extraction of the entire sample material. LAESI-TOF-MSI may provide a simplified alternative for qualitative analysis of pesticides on cut flowers. Here we analyzed the leaves of the cut flowers and searched for the presence of targeted pesticide residues from a shortlist of frequently occurring substances on cut flowers in The Netherlands. In Fig. 1 accurate ion maps are shown for (a) *m*/*z* 282.271 (±10 mDa), (b) *m*/*z* 298.265 (±10 mDa), and (c) *m*/*z* 317.158 (±10 mDa), superimposed on a camera image of the rose leaf. The mass measurements of the [M+H]^+^ ions are within 7–10 mDa of the theoretical values of the elemental compositions C_18_H_36_NO, C_18_H_36_NO_2_, and C_13_H_25_N_4_O_3_S, and suggest the presence of the pesticides dodemorph, spiroxamine, and bupirimate, respectively, in accordance with observations from previous analyses by GC–MS and LC–MS. Note that spiroxamine is a non-registered pesticide for roses. The similar spatial distribution of these different pesticides on a leaf surface strongly suggests a co-application of regulated and non-regulated pesticides, which is highly relevant information for agriforensics and enforcement. Of course, such an agriforensics case should be underpinned further by the analysis of a range of leaves from different cut flowers and include additional accurate mass MS–MS data.

**Fig. 2:**
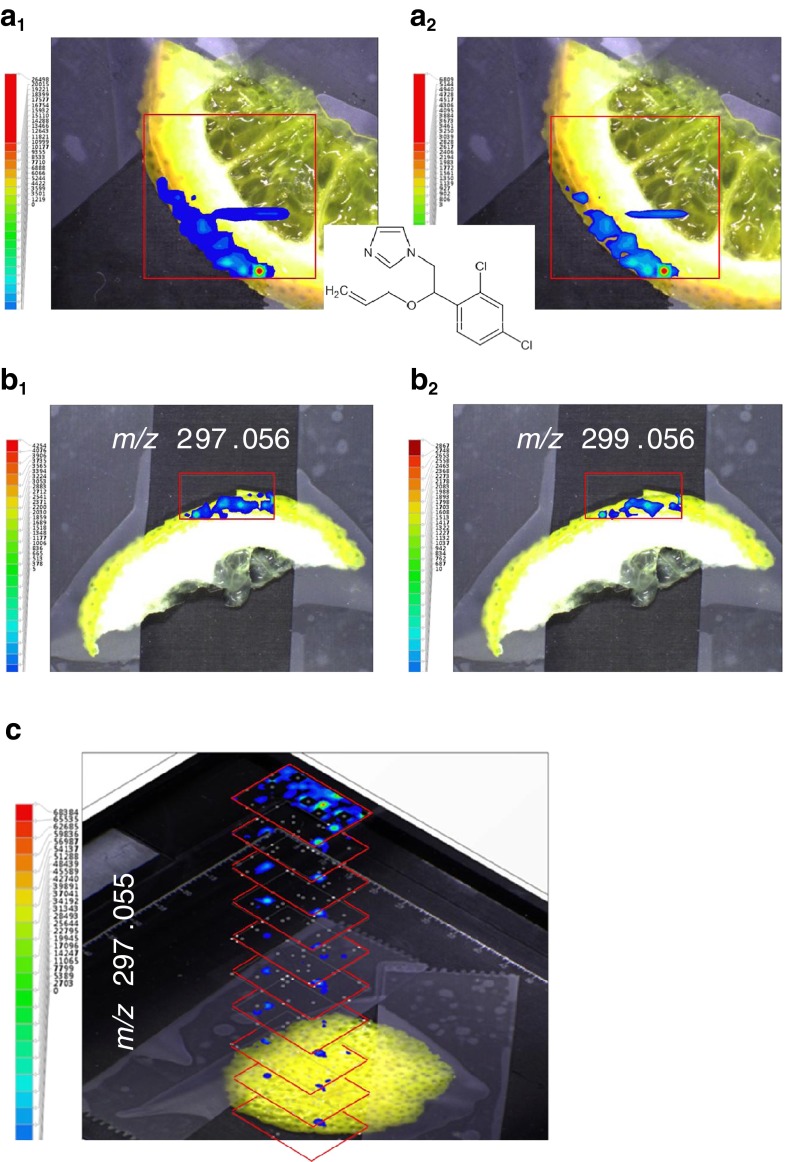
Positive-ion LAESI-TOF-MSI accurate ion maps of (**a**_**1**_, **b**_**1**_) the ^35^Cl^35^Cl isotope [M+H]^+^ ion at *m*/*z* 297.056 (±5 mDa), and (**a**_**2**_, **b**_**2**_) the ^35^Cl^37^Cl isotope [M+H]^+^ ion at *m*/*z* 299.056 (±5 mDa), on (**a**) orange slice and (**b**) lemon slice, showing the spatial distributions of the post-harvest fungicide imazalil. (**c**), 3D profiling of imazalil in a lemon-peel slice, represented by a stack of 2D ion maps of *m*/*z* 297.055; for details see text. The *x*–*y* center-to-center distance was 1 mm. Other conditions, see Fig. 1

Next, we analyzed slices of citrus fruit in 2D and 3D by LAESI-TOF-MSI for the presence and the penetration of the post-harvest fungicides imazalil and thiabendazole in the peel. Thiabendazole was absent but, in contrast, imazalil was found on all regular citrus fruit samples analyzed. In Fig. 2 accurate ion maps of *m*/*z* 297.056 (±5 mDa) and *m*/*z* 299.056 (±5 mDa) are shown for orange (Fig. 2a) and lemon slices (Fig. 2b) revealing the [M+H]^+^ ions of the ^35^Cl^35^Cl and ^35^Cl^37^Cl isotopes of imazalil. C_14_H_15_N_2_OCl_2_ is the first theoretical elemental-composition option corresponding with the accurate mass measurement and the Cl_2_ isotope pattern observed. The ion maps of both isotopes are very similar and provide an additional confirmation of identity. As can be seen from the superimposed figures, the fungicide is located on the peel only (except for the cutting artefact in Fig. 2a). Next we performed a 3D LAESI experiment by firing 10 individual laser shots on each *x*–*y* location at 1 Hz and correlating the MS data thus obtained not only by location but also by the shot number. As a result a 3D profile can be obtained represented by a stack of individual 2D accurate-mass ion images superimposed on the camera image of the slice (Fig. 2c). The 3D profile confirms that imazalil is mainly present in the first layers and a gradual decrease is observed from the top (sample surface) to deeper layers. According to literature [7, 11], the penetration depth per laser shot is in the order of 40 μm, but highly dependent on the tensile strength of the sample surface. Provided the literature estimate applies here, imazalil residues are not yet absent at 400 μm below the peel surface. Apple slices were also analyzed by LAESI-TOF-MSI to investigate the presence of diphenylamine residues. Diphenylamine is used as a pre or postharvest scald inhibitor for apples but this pesticide, although occasionally reported on apples from non-EU countries, was absent on the apples investigated here, and only ion maps of natural sugars were obtained. Note that spiked apple slices did reveal the accurate mass of the [M+H]^+^ ion of diphenylamine, within 5 mDa of its theoretical exact mass (Electronic Supplementary Material Fig. S2).

### Targeted spatially-resolved analysis of mycotoxins

Mycotoxins are secondary metabolites from fungi and expected to be more present at the surface of cereals. After fungal infection of rye by *Claviceps purpurea,* the seed is replaced by a hard dark-colored wintering body (“ergot body” or “ergot sclerotium”) that contains toxic ergot alkaloids. The types of ergot alkaloids and their corresponding epimers found will depend on the specific fungus, the plant, and local conditions. Usually, ergot body samples are ground, extracted, cleaned-up, and analyzed by liquid chromatography–tandem mass spectrometry (LC–MS–MS) [28]. Here we analyzed ergot bodies from rye, without any sample preparation or chromatography, using LAESI-TOF-MSI at a center-to-center distance of 200 μm. In a first rye sample, [M+H]^+^ ions of ergine + erginine, ergometrine + ergometrinine, ergotamine + ergotaminine, and ergocristine + ergocristinine were found on the ergot bodies. The diameters of the ablated craters were smaller than 200 μm and estimated to be actually 150 μm (Electronic Supplementary Material Fig. S3). According to these preliminary results, ergine + erginine was much more abundant than the other alkaloids and the edge and the groove of the ergot body were the preferred locations for all ergot alkaloids observed. Ergocornine + ergocorninine and ergocryptine + ergocryptinine were not observed in the ergot bodies from this particular rye sample. In contrast with LC–MS–MS [28], the epimers cannot be easily resolved by LAESI-TOF-MSI. It is possible that LAESI-TWIM-TOF-MSI could differentiate between these isomeric alkaloid pairs, but achieving this would be extremely challenging. For example, the theoretical collision-cross-section (CCS) values of single-charged protonated ergotamine and ergotaminine ions, as calculated by MMFF94s energy minimization using Avogadro software [29], differ by only 2 Å^2^. Note that the relatively high abundance of ergine + erginine was unexpected: in historical LC–MS–MS data on ergot bodies from our lab the levels of ergine + erginine are usually very low, which raises the question of whether decomposition of ergot alkaloids towards ergine + erginine occurred during LAESI-TOF-MSI. To test that hypothesis, microliter droplets of a 100 ppm ergotamine + ergotaminine standard were spiked onto the surface of a blank ergot body and analyzed at different laser frequencies (0.5, 1, and 10 Hz) and laser fluences (80 % and 100 %). Lowering the laser fluence caused a significant loss of sensitivity and was not an option; in contrast, lowering the pulse frequency caused an increase in the ergotamine + ergotaminine versus the ergine + erginine signal, but the latter remained by far the most abundant. Replacing the electrospray ionization solvent with methanol–water (1:1) without formic acid enabled analysis of the spiked blank ergot with improved ergotamine + ergotaminine signal intensities, being approximately two-thirds of the ergine + erginine signal. From these experiments, it was concluded that decomposition towards ergine + erginine could not be ruled out completely in our LAESI-MSI set-up. Future comparisons with liquid-extraction surface analysis (LESA) or nano-DESI should elucidate whether laser-induced thermal heating is a main contributor to these decomposition phenomena.

**Fig. 3:**
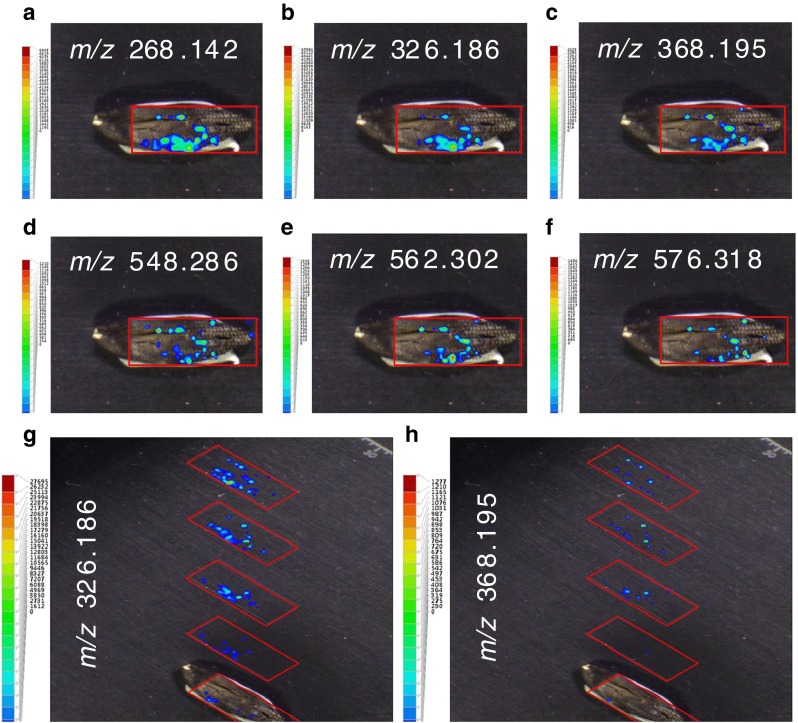
Positive-ion LAESI-TOF-MSI accurate ion maps of (**a**) *m*/*z* 268.142 (±5 mDa), (**b**) *m*/*z* 326.186 (±5 mDa), (**c**) *m*/*z* 368.195 (±5 mDa), (**d**) *m*/*z* 548.286 (±5 mDa), (**e**) *m*/*z* 562.302 (±5 mDa), and (**f**) *m*/*z* 576.318 (±5 mDa) on an ergot body from rye, showing the spatial distribution of the [M+H]^+^ ions of the ergot alkaloids ergine+erginine, ergometrine+ergometrinine, an untargeted alkaloid, ergosine+ergosinine, ergocornine+ergocorninine, and ergocryptine+ergocryptinine, respectively. (**g**) and (**h**) 3D profiling of ergometrine+ergometrinine and the untargeted ergoval, represented by a stack of 2D ion maps of *m*/*z* 326.186 (±5 mDa) and *m*/*z* 368.195 (±5 mDa); for details see text. The *x*–*y* center-to-center distance was 0.3 mm. For other conditions, see Fig. 1

Next, another ergot body from a different rye sample was analyzed using LAESI-TOF-MSI at a center-to-center distance of 300 μm, methanol–water (1:1) as spray solvent, and a mid-IR laser setting of 100 % at 1 Hz; the results are shown in Fig. 3a–f. The experimentally obtained [M+H]^+^ ions are within 0–2 mDa of the theoretical values of ergine + erginine, ergometrine + ergometrinine, ergosine + ergosinine, ergocornine + ergocorninine, and ergocryptine + ergocryptinine. As can be seen in Fig. 3a–f, all ergot alkaloids are co-localized and ergine + erginine and ergometrine + ergometrinine are the most abundant in these LAESI experiments. The former pair may be at least partly caused by decomposition during the experiment (cf. above) but the ergometrine + ergometrinine pair was not observed as a decomposition product during the experiments with spiked ergotamine and therefore most probably indicates the actual presence of ergot alkaloids. The ion map of *m*/*z* 368.195 (Fig. 3c) is from an untargeted substance having an odd number of nitrogen atoms. Within 5 mDa only the elemental compositions C_21_H_26_N_3_O_3_ and C_16_H_26_N_5_O_5_ are solutions, and from these options only C_21_H_26_N_3_O_3_ would have a sufficient rings-plus-double-bonds number to be an ergot-alkaloid-related compound, as clearly suggested by its co-localization. Following a Scifinder elemental composition plus substructure search, ergoval (lysergyl-valine) was suggested as a candidate. This substance has been reported very rarely in literature but has been identified in fungal extracts by nanoLC–MS–MS [30]. Finally, 3D profiles of ergometrine + ergometrinine and ergoval were recorded (Fig. 3g–h), revealing a gradual decrease in ergot alkaloid intensities from top (sample surface) to deeper layers, in accordance with expectations for fungal secondary metabolites.

### Untargeted spatially-resolved analysis of natural components

**Fig. 4:**
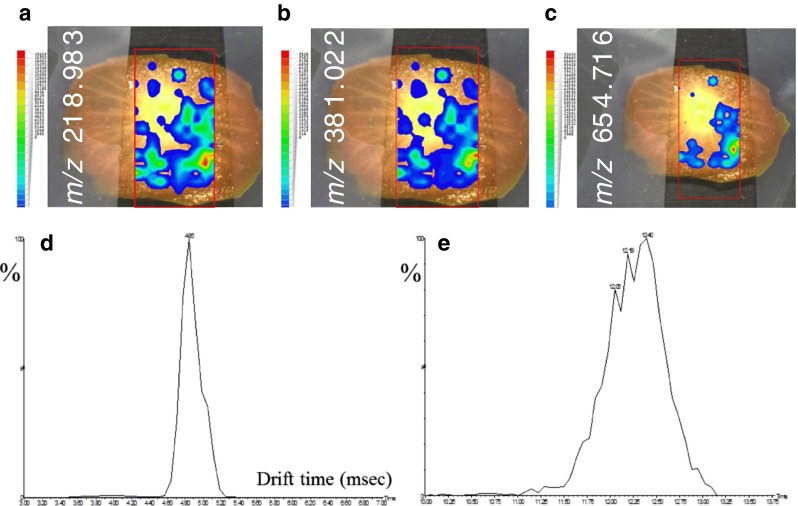
Untargeted positive-ion LAESI-TWIM-TOF-MSI ion maps of (**a**) *m*/*z* 218.983 (±5 mDa), (**b**) *m*/*z* 381.022 (±5 mDa), and (**c**) *m*/*z* 654.716 (±5 mDa) for a cherry-tomato slice, showing the spatial distribution of the natural carbohydrates C_6_H_12_O_6_, C_12_H_22_O_11_ (both [M+K]^+^ ions), and lycoperosides F and/or G and/or esculeoside A ([M+H+K]^2+^ ion). The *x*–*y* center-to-center distance was 0.5 mm. (**d**) and (**e**) reconstructed ion-drift time distributions for lycoperosides F and/or G and/or esculeoside A, showing the [M+H+K]^2+^ ion at *m*/*z* 654.716 (±5 ppm) and the [M+H]^+^ ion at *m*/*z* 1270.509 (±5 ppm), respectively. No lock-mass correction performed. For other conditions see Fig. 1

Ambient MS of foods is usually performed in a targeted manner for rapid analysis of, e.g., pesticide residues on fruit and vegetables [25, 26] or for profiling of specific vegetable oils [31]. Spatially-resolved information may be very relevant to localize potential healthy or harmful natural components and study their origin and fate. Here we performed ambient imaging MS of cherry-tomato slices using LAESI-TOF-MSI and LAESI-TWIM-TOF-MSI. A major difference from the investigations above was the power setting of the mid-IR laser: because of the water-rich nature of tomatoes a setting of 30 % is adequate for generating an ablation plume. Higher laser fluences not only yield wide and deep ablation craters but also cause severe surface contamination, thereby diminishing the integrity of the spatially-resolved chemical information. The mass spectra and reconstructed ion chronograms obtained indicated the presence of a range of common carbohydrate ions including [C_6_H_12_O_6_ + Na and K]^+^, its [2M + Na]^+^ ion, [C_12_H_22_O_11_ + K]^+^, and [C_18_H_30_O_17_ + K]^+^. Interestingly, two double-charged ions were observed, at *m*/*z* 646.721 and *m*/*z* 654.716, that must contain an odd number of nitrogen atoms and probably indicate the presence of the [M+H+Na]^2+^ and [M+H+K]^2+^ ions of one or more of the steroidal glycoalkaloids lycoperoside F or G or esculeoside A. In Fig. 4a–c ion maps of (a) *m*/*z* 218.983 (±5 mDa), (b) *m*/*z* 381.022 (±5 mDa), and (c) *m*/*z* 654.716 (±5 mDa) are given that clearly show the co-localization of common carbohydrates and the steroidal glycoalkaloid(s). Lycoperoside F was reported previously as the [M+H]^+^ ion at *m*/*z* 1270.5 in a study by Peng et al. in which they manually inserted nano-electrospray capillaries into tomatoes and plants for localized analysis of bioactives [32]. As well as the high-intensity double-charged ions, low-intensity [M+H]^+^ ions were also observed at *m*/*z* 1270.5 in our LAESI study. However, three different lycoperoside and esculeoside isomers exist, lycoperoside F and G and esculeoside A, which have an identical elemental composition to C_58_H_95_NO_29_. Drift-time distributions were reconstructed for the [M+H+K]^2+^ ion at *m*/*z* 654.716 (±5 ppm) and the [M+H]^+^ ion at 1270.509 (±5 ppm) in a preliminary attempt to differentiate between the isomeric steroidal glycoalkaloids (Fig. 4d, e). In the double-charged-ion drift-time distribution (Fig. 4d) one main peak and a lower shoulder peak can be observed, the latter having a longer drift time and probably a larger experimental CCS value; the separation in the single-charged-ion drift-time distribution (Fig. 4e) seems to be slightly better but actually the signal-to-noise ratio of this ion is rather low. The theoretical CCS values of single-charged protonated lycoperoside F and G and esculeoside A ions, as calculated by MMFF94s energy minimization using Avogadro software [29], are 271, 256, and 268 Å^2^, respectively. Assuming that a similar theoretical CCS trend would apply to the double-charged [M+H+Na]^2+^ ions in Fig. 4d, the main peak is probably lycoperoside G and the lower shoulder lycoperoside F and/or esculeoside A. More research, including ion-mobility QTOF-MS–MS studies using pure reference standards, is needed to fully resolve the isomeric structures of these glycoalkaloids with the same exact mass.

**Fig. 5:**
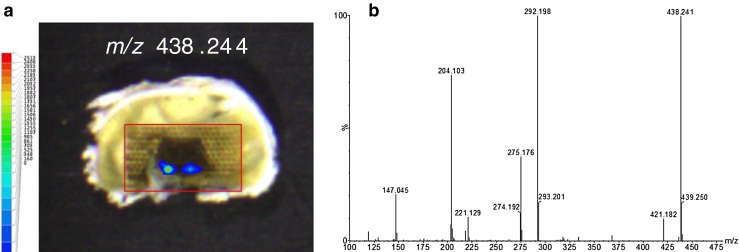
Untargeted positive-ion LAESI-QTOF-MS–MS ion map of (**a**) *m*/*z* 438.244 (±5 mDa) on a tip cap of a maize kernel, showing the spatial distribution of an unknown. The *x*–*y* center-to-center distance was 0.2 mm. (**b**) centroided MS–MS spectrum of precursor ion *m*/*z* 438.2 (the MS–MS spectrum was lock-mass corrected after the data acquisition using the precursor ion *m*/*z* 438.241)

As a second example of untargeted ambient imaging, LAESI-TOF-MSI and LAESI-QTOF-MS–MS were applied to tip caps of maize kernels. Two different types of maize kernel were investigated: normal ones, and suspect kernels with darker areas in the tip cap of the kernel. The mass spectra and reconstructed ion chronograms obtained suggested the presence of a range of low-molecular-weight ions, including such common plant metabolites as a dihydroxycinnamic acid (e.g. caffeic acid), a trihydroxybenzoic acid (e.g. gallic acid), a hydroxybenzoic acid, and a dihydroxybenzoic acid. Interestingly, the tip-cap regions of both maize-kernel types also produced two highly localized signals having different relative intensities: an [M+Na]^+^ ion at *m*/*z* 463.183 and an [M+H]^+^ ion at *m*/*z* 438.241. Assuming an elemental composition containing only C, H, and O atoms, the [M+Na]^+^ ion at *m*/*z* 463.183 would correspond with the composition C_18_H_32_O_12_ (mass error 3.9 mDa). Several C_6_ fatty esters of disaccharides have that composition, among them sucrose caproate, which has been reported as a seed-germination inhibitor [33]. As expected, MS–MS experiments on the [M+Na]^+^ ion were not successful and consequently we were unable to discriminate between the different fatty-ester disaccharide options. The spatial distribution of the [M+H]^+^ ion at *m*/*z* 438.241 is shown in Fig. 5a. Note that this even-mass [M+H]^+^ ion must contain an odd number of nitrogen atoms. Assuming an elemental composition of C, H, N, and O atoms, many options are possible for this ion within a mass error of 5 mDa. The LAESI-QTOF-MS–MS spectrum of the [M+H]^+^ precursor ion *m*/*z* 438.2 is shown in Fig. 5b. Using this MS–MS spectrum, elemental compositions of fragment ions and neutral losses were calculated and (sub)structures proposed (Table 1). All assigned elemental compositions are within 0–6 mDa of their theoretical value. The ion at *m*/*z* 292.198 is directly formed from the precursor ion and fragments further, with neutral losses of 17.022, 74.087, and 88.095 Da corresponding with NH_3_, C_3_H_10_N_2_, and C_4_H_12_N_2_, respectively. Therefore, the unknown compound with an even-mass [M+H]^+^ ion does not contain only a single nitrogen but should contain (at least) three nitrogen atoms. Together with the neutral loss of 57.058 Da, a spermidine substructure is clearly suggested. Spermidine is a polyamine plant-growth regulator that occurs in, among others, corn. The neutral loss of 146.043 Da and the fragment ion at *m*/*z* 147.045 both support coumaroyl-like substructures. Altogether, *N*,*N*-bis-coumaroyl spermidine is a strong candidate for the identity of the highly localized unknown compound with an [M+H]^+^ ion at *m*/*z* 438.241. *p*-Coumaric acid amides of polyamines have been associated with the chemical defense of plants against arthropod pests. These compounds were found to be bioactive at the receptor level but not when ingested [34].

**Table 1:**
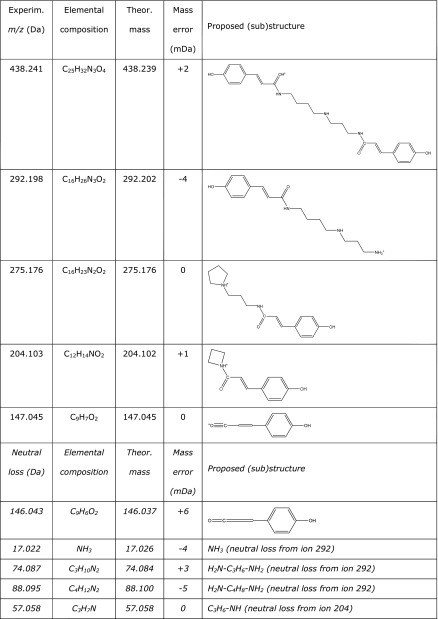
Elemental compositions of ions and neutrals losses in the MS–MS spectrum in Fig. 5b, and tentatively proposed (sub) structures

## Conclusion

In these investigations, LAESI-MSI of rose leaves, orange, apple, and lemon fruit, ergot bodies, cherry tomato, and maize kernels was revealed to be straightforward. The large sample stage enabled both macroscopic and microscopic imaging, no precision sample slicing was required, no MALDI matrix had to be added, and the analyses could be performed under ambient ionization conditions. In many cases macroscopic spatial resolution will be fit for purpose, for example when studying the co-application and/or the penetration of pesticides into fruit and vegetables. In other situations, microscopic imaging will be required to localize the presence of harmful natural toxins more precisely, not only for food quality and safety decisions but also to support further research into the formation and fate of natural toxins. The ease of use of LAESI-MSI is in contrast with alternative DESI, nanoDESI, and MALDI imaging studies of cell cultures, potato, rice, and beef [15, 35–37]. However, some LAESI-specific disadvantages should also be mentioned. The mid-IR ablation at 2,940 nm mainly relies on the presence of water in the sample, so an inhomogeneous water distribution may affect the effective ablation of neutrals. As a result, fewer molecules may be ionized, thereby suggesting lower levels locally than are actually present. The same holds for local changes in tensile strength. The spatial resolution of LAESI is lower than that of SIMS, but recent developments reveal LAESI performances equaling MALDI-MSI [20]. Other more general MSI-related difficulties apply equally to LAESI-MSI of foods, and challenges remain with respect to: full understanding of desorption and ionization processes, obtaining more quantitative imaging data, and handling the immense data files generated (including fast visualization software solutions). It has recently been stated that MSI is not suitable for discovery purposes and only applicable to targeted imaging of known molecules, because optimization of experimental conditions is highly dependent on the properties of the analytes [2]. In general, targeted MSI is indeed the most straightforward, including in terms of data handling, but from our untargeted LAESI-MSI examples (Figs. 3c, 4c, and 5b) we have revealed that valuable spatially-resolved chemical information can also be obtained without a-priori knowledge. Therefore, it is concluded here that LAESI-MSI combined with ion mobility and MS–MS features may contribute to the identification of unexpected and unknown chemical features in food products and food ingredients.

## Electronic supplementary material

Below is the link to the electronic supplementary material.

ESM 1 (PDF 239 kb)
