# Supplementary material for: Macroscopic and microscopic spatially-resolved analysis of food contaminants and constituents using laser-ablation electrospray ionization mass spectrometry imaging
Source: Anal Bioanal Chem. 2014 Jun 25;406(27):6805–15. doi: 10.1007/s00216-014-7948-8 (PMC4196196; doi:10.1007/s00216-014-7948-8)

Analytical and Bioanalytical Chemistry

Electronic Supplementary Material

Macroscopic and microscopic spatially-resolved analysis of food contaminants and constituents using laser-ablation electrospray-ionization mass-spectrometry imaging

Michel W.F. Nielen, Teris A. van Beek

Figure S1 Schematic overview of the laser ablation electrospray ionization time-of-flight mass spectrometry imaging (LAESI-TOF-MSI) set-up.

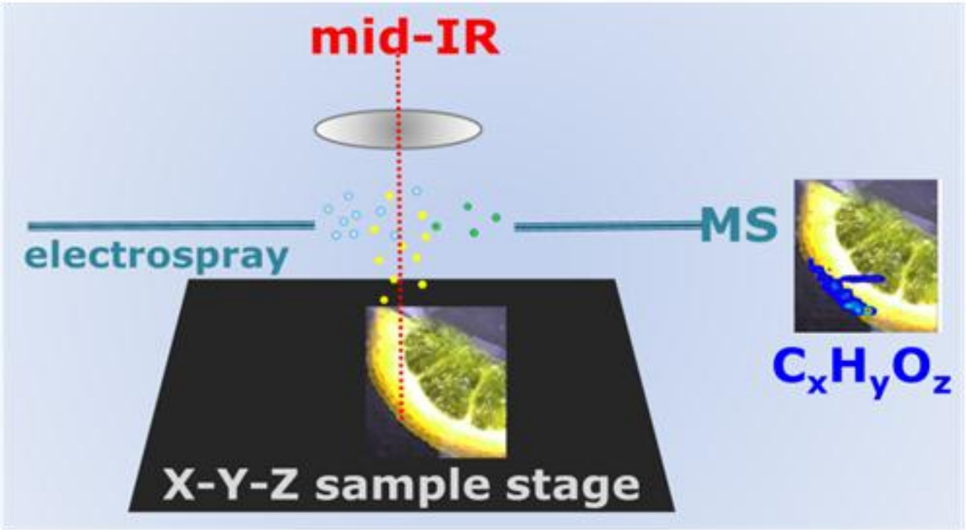

Figure S2 Positive ion LAESI-TOF-MSI accurate ion maps of (**a**) *m/z* 203.038 (±10 mDa) and (**b**) *m/z* 365.101 (±10 mDa) on a fresh apple slice, showing the spatial distribution of the $[M+Na]^+$ ions of the sugars $C_6H_{12}O_6$, and $C_{12}H_{22}O_{11}$, respectively. No traces of the banned scald inhibitor diphenylamine ($[M+H]^+$ ion expected at m/z 170.097) were found on the peel. (**c**) Apple slice spiked with diphenylamine did show the pesticide within 5 mDa of its theoretical exact mass. The x-y center-to-center sampling distance was 1 mm. The probed region is indicated by a red rectangular; the lowest ion intensities are not color-coded to enable visualization of the underlying features in the superimposed sample image. Other conditions, see *Experimental section*.

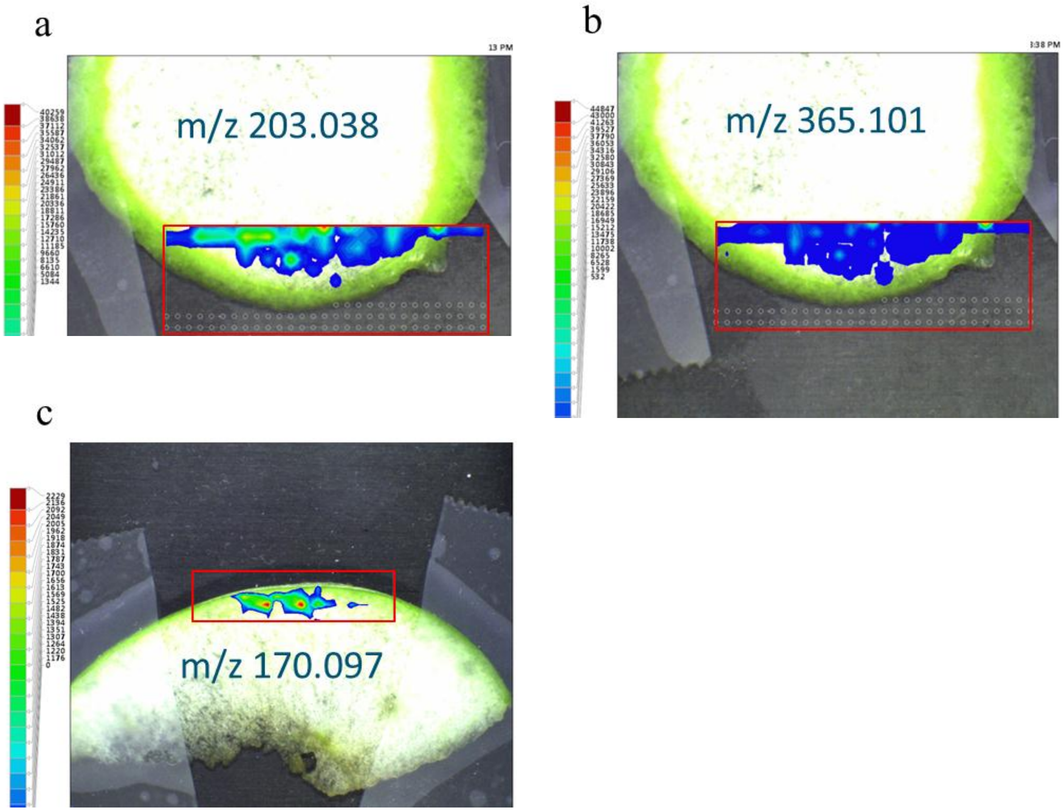

Figure S3 Positive ion LAESI-TOF-MSI mass spectra showing the $[M+H]^+$ ions of (**a**) ergine+erginine, (**b**) ergometrine+ ergometrinine, (**c**) ergotamine+ergotaminine and (**d**) ergocristine+ergocristinine from specific locations on an ergot body from rye. (**e**) Positive ion LAESI-TOF-MSI accurate ion map of *m/z* 268.142 (±5 mDa) on an ergot body, showing the spatial distribution of the $[M+H]^+$ ions of ergine+erginine. The x-y center-to-center distance was 200 μm. Other conditions, see Experimental section.

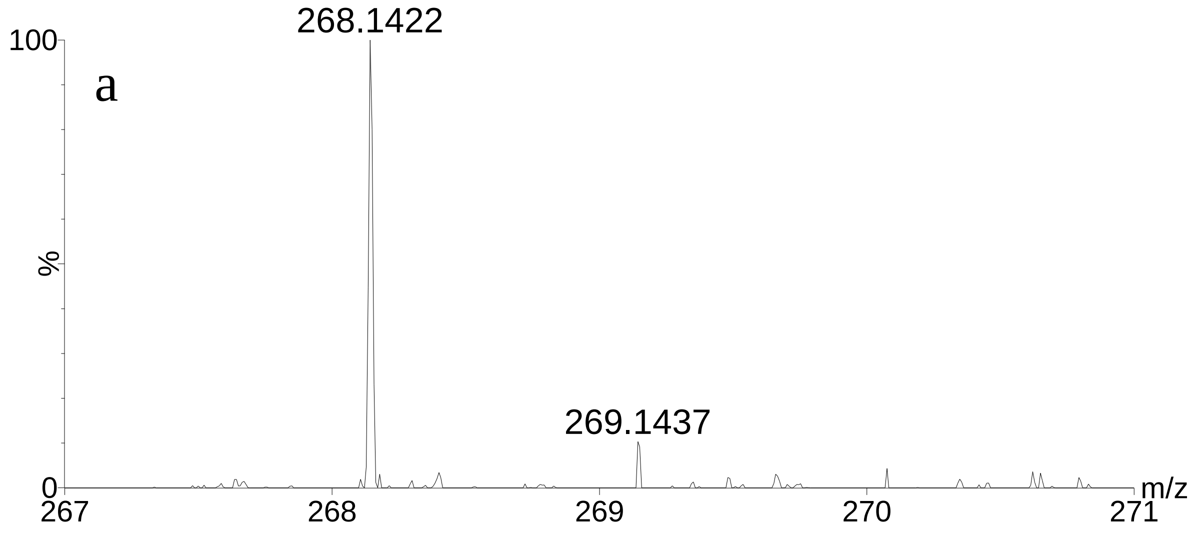

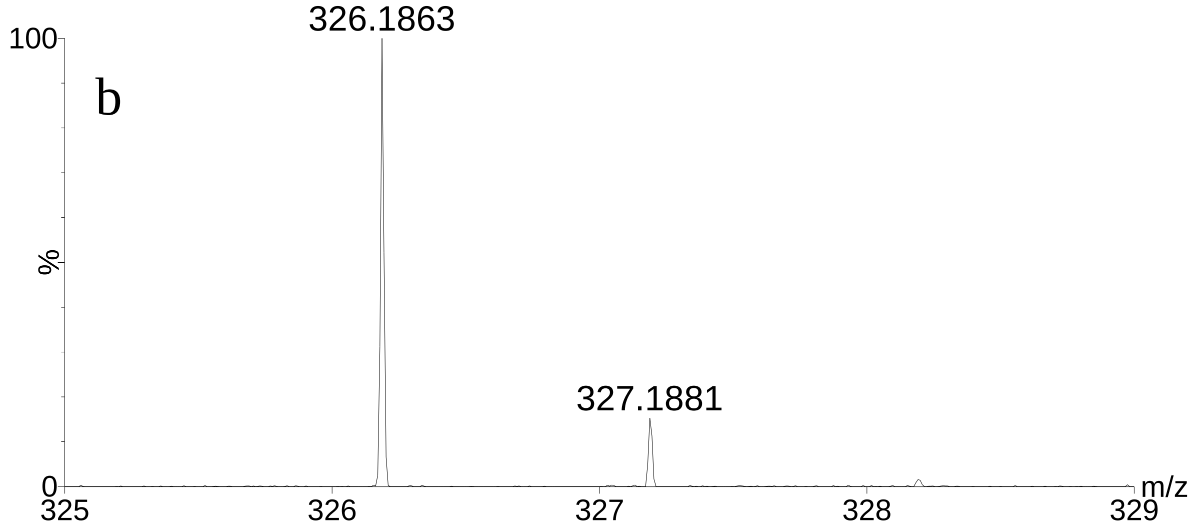

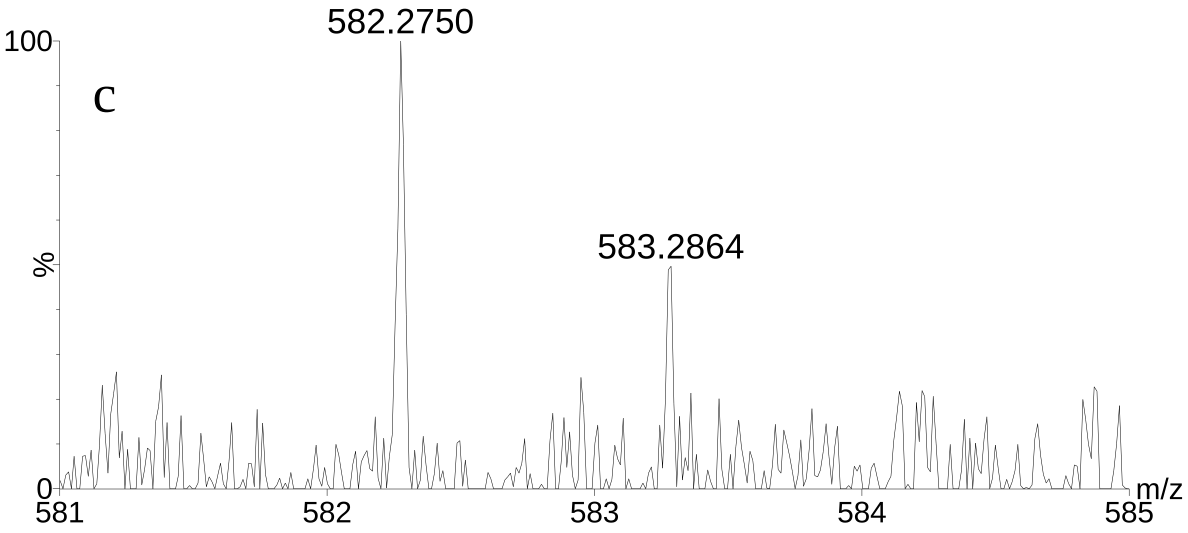
582.2750
583.2864
100
%
0
m/z
581
582
583
584
585
c

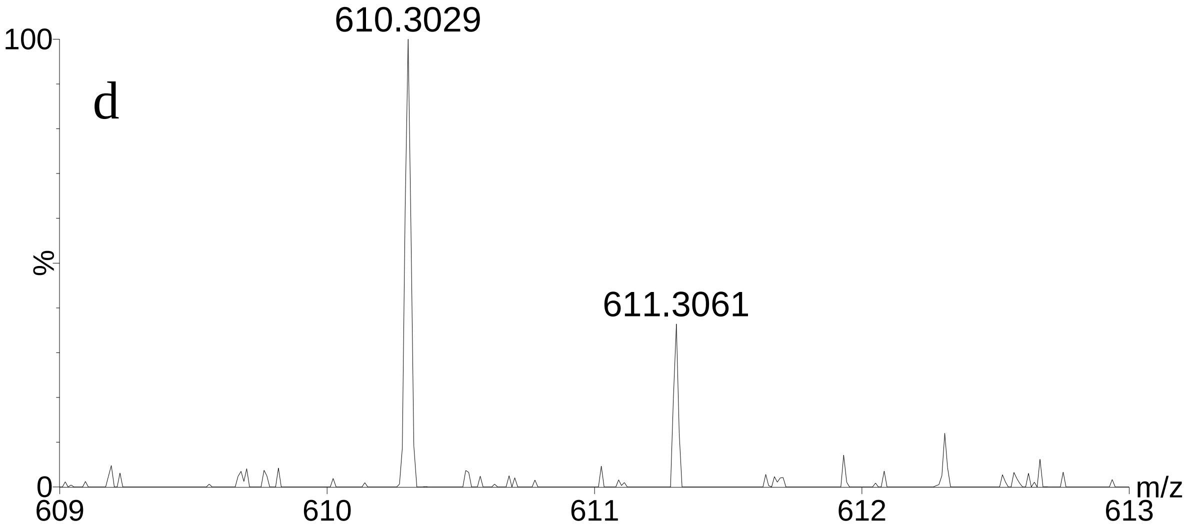
610.3029
611.3061
100
%
0
m/z
609
610
611
612
613
d

e

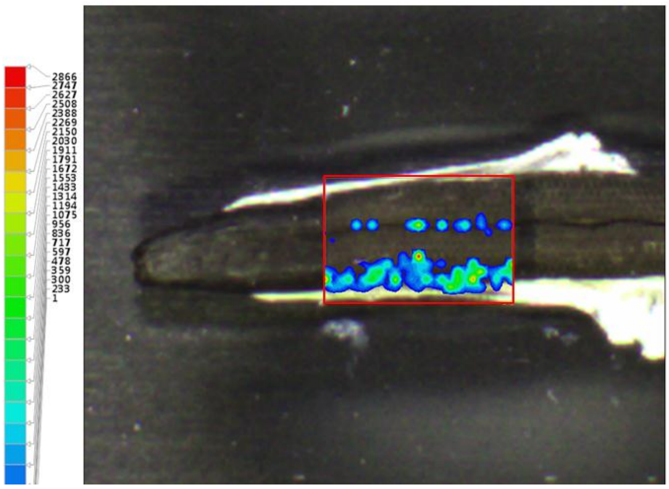

Supplement: Supplementary file 1 — (PDF 239 kb) [file 216_2014_7948_MOESM1_ESM.pdf]
